# Temporal Trends of System of Care for STEMI: Insights from the Jakarta Cardiovascular Care Unit Network System

**DOI:** 10.1371/journal.pone.0086665

**Published:** 2014-02-10

**Authors:** Surya Dharma, Bambang Budi Siswanto, Isman Firdaus, Iwan Dakota, Hananto Andriantoro, Alexander J. Wardeh, Arnoud van der Laarse, J. Wouter Jukema

**Affiliations:** 1 Department of Cardiology and Vascular Medicine, Faculty of Medicine, University of Indonesia, National Cardiovascular Center Harapan Kita, Jakarta, Indonesia; 2 Department of Cardiology, M.C. Haaglanden, The Hague, The Netherlands; 3 Department of Cardiology, Leiden University Medical Center, Leiden, the Netherlands; 4 Interuniversity Cardiology Institute the Netherlands, Utrecht, the Netherlands; University Heart Center, Germany

## Abstract

**Aim:**

Guideline implementation programs are of paramount importance in optimizing acute ST-elevation myocardial infarction (STEMI) care. Assessment of performance indicators from a local STEMI network will provide knowledge of how to improve the system of care.

**Methods and Results:**

Between 2008–2011, 1505 STEMI patients were enrolled. We compared the performance indicators before (n = 869) and after implementation (n = 636) of a local STEMI network. In 2011 (after introduction of STEMI networking) compared to 2008–2010, there were more inter-hospital referrals for STEMI patients (61% vs 56%, p<0.001), more primary percutaneous coronary intervention (PCI) procedures (83% vs 73%, p = 0.005), and more patients reaching door-to-needle time ≤30 minutes (84.5% vs 80.2%, p<0.001). However, numbers of patients who presented very late (>12 hours after symptom onset) were similar (53% vs 51%, NS). Moreover, the numbers of patients with door-to-balloon time ≤90 minutes were similar (49.1% vs 51.3%, NS), and in-hospital mortality rates were similar (8.3% vs 6.9%, NS) in 2011 compared to 2008–2010.

**Conclusion:**

After a local network implementation for patients with STEMI, there were significantly more inter-hospital referral cases, primary PCI procedures, and patients with a door-to-needle time ≤30 minutes, compared to the period before implementation of this network. However, numbers of patients who presented very late, the targeted door-to-balloon time and in-hospital mortality rate were similar in both periods. To improve STEMI networking based on recent guidelines, existing pre-hospital and in-hospital protocols should be improved and managed more carefully, and should be accommodated whenever possible.

## Introduction

The recent 2012 European Society of Cardiology (ESC) guideline on ST-segment elevation myocardial infarction (STEMI) stressed the importance of networking for the management of acute myocardial infarction (AMI) [1]. In an earlier report, we emphasized the concept of a trained health system network in order to decrease the mortality rate of STEMI patients. The mission of such a network is how to increase the use of acute reperfusion treatment in the pre-hospital and hospital settings, using a pharmaco-invasive strategy in Jakarta, Indonesia [2].

After the initial introduction of the network, we analyzed the effectiveness of the system to improve the network protocols using a registry that we set up in 2008 as an integral part of modern health care [3], [4]. We analyzed the quality of care and performance indicators of our local acute coronary syndrome registry, to further improve the STEMI system of care in Jakarta, Indonesia.

## Methods

**Figure 1:**
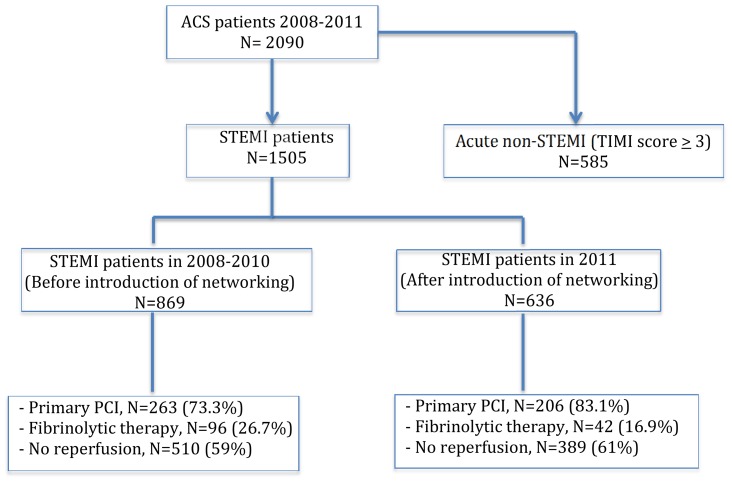
Patient distribution in the Jakarta Acute Coronary Syndrome registry. ACS = acute coronary syndrome, STEMI = ST-elevation myocardial infarction, PCI = percutaneous coronary intervention, TIMI = Thrombolysis in Myocardial Infarction.

Data was collected from the Jakarta Acute Coronary Syndrome (JAC) registry database which included 1505 patients admitted with acute STEMI in the Emergency Department of the National Cardiovascular Center Harapan Kita (NCCHK), Jakarta, Indonesia from 2008 to 2011 (Figure 1). The NCCHK acts as a national referral hospital and the main receiving center in Jakarta with 24 hours cardiovascular services including primary PCI capabilities. Initial diagnosis was made on the basis of presence of typical chest pain and ST segment elevation (≥0.1 mV) in two or more contiguous leads on the admission ECG.

All demographic, clinical and laboratory variables were obtained from a standardized STEMI registry form. Raised body mass index (BMI) was defined as a BMI>25 kg/m^2^. Diabetes mellitus was diagnosed in patients with a history of oral antidiabetic or insulin medication or fasting blood glucose >125 mg/dl at study entrance; hypertension was diagnosed by the Joint National Committee VII criteria on hypertension or if currently taking antihypertensive treatment; dyslipidemia was diagnosed in patients with a history of lipid lowering medication or a fasted total cholesterol level >200 mg/dl, or LDL >130 mg/dl, or HDL<40 mg/dl, or triglyceride >150 mg/dl, and a positive family history of premature coronary artery disease (CAD) if CAD had developed before the age of 65 years in a first degree relative.

We compared the profiles of STEMI patients before the introduction of the STEMI networking (between 2008–2010) with those after introduction of the network (in 2011). Reperfusion therapy was given according to the recommendations of the ESC and American College of Cardiology/American Heart Association guidelines. The JAC registry and the analysis of the registry described in this manuscript have been approved by the institutional review board (IRB) committee of the Department of Cardiology and Vascular Medicine, Faculty of Medicine, University of Indonesia, National Cardiovascular Center Harapan Kita. There is no informed consent because data were analyzed anonymously and waived by the IRB committee.

### Study endpoint

Study endpoints are the performance indicators in two time periods: before and after the implementation of the network, such as the number of inter-hospital referral cases, number of primary PCI procedures, number of patients who presented very late (>12 hours after onset of chest pain), and the time delay between admission to the hospital and actual reperfusion (door-to-balloon time and door-to-needle time).

### Statistical methods

Continuous variables are presented as mean values ± standard deviation (SD) or median (minimum-maximum) if not fitting a normal distribution. Categorical variables were expressed as percentages or proportions. Normally distributed variables were compared by Student *t*-test and skewed distribution data by Mann-Whitney U-test. Categorical variables were tested by chi-square test. A p value<0.05 was considered significant. All statistical analyses were performed with SPSS version 17.0 (SPSS Inc., Chicago, IL, USA).

## Results

The median age of the STEMI patients was 55 years (ranging from 24 to 96 years) and the majority was male (86%). As reported earlier [2], hypertension was the most common risk factor (54%) in our STEMI population and the risk factors did not differ between the two periods. The source of referral was mostly from another hospital (58.3%) (Table 1).

**Table 1:** Demographic data and hospitalization information of STEMI patients (N = 1505).

Variables	Description
Age, years	55 (24–96)
Gender, N (%)	
Female	214 (14,2%)
Male	1291 (85.7%)
Source of referral, N (%)	
Walk in/ambulance	502 (33.3%)
Primary physician	56 (3.7%)
Inter-hospital	878 (58.3%)
Intra-hospital	70 (4.6%)
Risk factor profile	
Raised BMI (>25 kg/m^2^)	320 (21.2%)
Carotid artery stenosis	3 (0.2%)
Family history of known CAD	368 (24.4%)
Dyslipidemia	580 (38.5%)
Hypertension	813 (54%)
Diabetes Mellitus	434 (29%)
Current smoker	698 (46.3%)

BMI = body mass index, CAD = coronary artery disease.

The number of inter-hospital referrals for STEMI patients has significantly increased in 2011 compared to 2008–2010 (61.2% vs 56.2%, p<0.001), but numbers of patients with STEMI who presented very late were similar (53.1% vs 51.2%, p = 0.466). There was a significant increase of primary PCI in 2011 (83.1% vs 73.3% p = 0.005) (Table 2). For patients who received fibrinolytic therapy, the numbers of patients with a door-to-needle time ≤30 minutes was higher in 2011 than in 2008–2010 (84.5% vs 80.2%, p<0.001). For patients who underwent primary PCI, the number of patients with a door-to-balloon time <90 minutes had not improved (49.1% vs 51.3%, p = 0.364) (Table 3). In-hospital mortality had not changed between 2011 and 2008–2010 (8.3% vs 6.9%, p = 0.303).

**Table 2:** STEMI profile based on network application period.

Variables	2008–2010 (before implementation of AMI networking)	2011 (after implementation of AMI networking)	P value
	N = 869	N = 636	
Age, years	55 (24–85)	55 (29–96)	0.407
Male, N (%)	735 (84%)	556 (87%)	0.242
Referral status			
Walk in/ambulance	281 (32.3%)	221 (34.7%)	
Primary physician	43 (4.9%)	13 (2.0%)	
Inter-hospital	488 (56.2%)	390 (61.2%)	<0.001
Intra-hospital	57 (6.6%)	13 (2.0%)	
Risk Factors			
Hypertension	457 (52.6%)	356 (56%)	0.339
Family History of known CAD	199 (22.9%)	169 (26.6%)	0.224
Dyslipidemia	305 (35.1%)	275 (43.2%)	0.202
Diabetes Mellitus	236 (27.2%)	198 (31.1%)	0.190
Current Smoker	399 (45.9%)	299 (47%)	0.806
Onset of infarction			
≤12 hours	422 (48.8%)	299 (46.9%)	0.466
>12 hours	442 (51.2%)	338 (53.1%)	
Reperfusion strategy			
Primary PCI	263 (73.3%)	206 (83.1%)	0.005
Fibrinolytic therapy	96 (26.7%)	42 (16.9%)	

Data are presented as numbers and percentages. PCI = percutaneous coronary intervention.

**Table 3:** Characteristics of STEMI patients before and after implementation of Jakarta Cardiovascular Care Unit Network System.

Variables	2008–2010 (before implementation of MI networking)	2011 (after implementation of MI networking)	P value
	N = 869	N = 636	
Location of MI			
Anterior	530 (61%)	376 (59.1%)	0.464
Non anterior	339 (39%)	260 (40.9%)	
Killip class			
I	598 (69.2%)	429 (68.5%)	
II	223 (25.8%)	151 (24.1%)	0.047
III	25 (2.9%)	17 (2.7%)	
IV	18 (2.1%)	29 (4.6%)	
DTN≤30 minutes	77 (80.2%)	120 (84.5%)	<0.001
DTB≤90 minutes	135 (51.3%)	105 (49.1%)	0.364
In-hospital mortality	60 (6.9%)	53 (8.3%)	0.303

Data are presented as numbers and percentages. MI = myocardial infarction, DTN = door-to-needle time, DTB = door-to-balloon time.

## Discussion

The Jakarta Cardiovascular Care Unit Network system was built to improve the system of care of AMI in Jakarta, Indonesia, serving about 11 million people with living in a density of 15,000 people/km^2^ [2]. The effectiveness of the system can be monitored by recording the performance indicators in STEMI patients, such as number of patients receiving acute reperfusion treatment (numbers of primary PCI and fibrinolytic therapy), time from door to reperfusion, and number of patients who presented very late [4], [5].

After the introduction of the network, there was a growing awareness of the primary physician in the primary hospital, as is shown by the increased numbers of STEMI patients referred from another hospital.

In the receiving center, the number of patients receiving primary PCI has increased after the application of the network, which might suggest that the pre-hospital protocol to make an accurate diagnosis of AMI has improved. However, the proportion of patients who received PCI with a door-to-balloon time ≤90 minutes, as recommended by the guideline, had not improved between the two periods. It has shown earlier that when PCI-related time delay increases, the mortality benefit decreases [6]–[8]. Moreover, the 2012 ESC guideline on management of STEMI patients [1] has strengthen the importance of shortening the time delay for primary PCI, and recommends a door-to-balloon time <60 minutes in a PCI-capable hospital.

The numbers of patients receiving fibrinolytic therapy have decreased in 2011, although more patients had reached a door-to-needle time ≤30 minutes compared to the 2008–2010 period before the network was introduced. It should be noted that fibrinolytic therapy of STEMI patients was given in the in-hospital setting. If the estimated first medical contact to balloon time is >120 minutes, we wish to start, in the near future, fibrinolytic therapy in the pre-hospital setting, as recommended by the guideline [1]. Local authorities have to collaborate in training all health care providers on how to perform fibrinolytic therapy according to a standard protocol.

Finally, the proportion of patients with STEMI who presented very late (>12 hours) had not improved between the two periods. As late presentation is associated with high mortality [2], we should get the patients to the hospitals that provide reperfusion therapy earlier. The in-hospital mortality has not changed in the two periods, which may be expected as the proportions of patients with door-to-balloon <90 minutes and patients who presented very late (>12 hours) have not changed between the two periods. For that purpose we have to analyse how to improve patient delay by recognizing the symptoms earlier and system delay by using electrocardiography (ECG) transmission and improving availability of ambulances.

Based on the results of the performance indicators before and after network introduction, the pre-hospital and in-hospital protocols should be improved. In pre-hospital protocols improvements to be implemented include: 1) the use of pre-hospital triage forms. We have made two models of a pre-hospital triage form and an ambulance communication chart form (Figures S1 and S2). These forms should be filled by the healthcare providers in the pre-hospital setting; 2) the 12-lead ECG should be recorded in all patients with suspected AMI and should be transmitted to our center as the host of the network. Pre-hospital 12-lead ECG plays an important role in a system of care for STEMI patients [9]–[11]. Currently, we are using a fax machine for ECG transmission but this system has several limitations, such as the unavailability of a fax machine in the ambulance. Therefore, we should transmit the ECG by a telephone- or, internet-based system; 3) a routine educational course should be attended to improve the skill and knowledge of the primary physician and nurses who are working in the emergency department or ambulance. To improve the in-hospital protocols of the receiving center, several improvements to implement are: 1) all administration processes related to reperfusion treatment should be managed in the emergency department as an integrated health care system; 2) as the host of the network, we have installed a catheterization laboratory in the emergency department that may contribute to reduce the time delay to reperfusion treatment; 3) for STEMI patients in whom the diagnosis has been made in the pre-hospital setting, the patients should be send directly to the cath-lab, by-passing the emergency department.

An intensive collaboration should be made between Indonesian Heart Association, Indonesian Heart Foundation and the local government of Jakarta in order to: 1) provide an education to the community about recognizing earlier signs and symptoms of a heart attack; 2) not to fear of coming to hospital; and 3) find the best solution for financial issues related to reperfusion therapy.

Prior AMI guideline implementation programs have improved patient care and patient's outcome [12]–[14]. However, the widespread dissemination of evidence-based medicine in daily practice is still lacking and a significant number of patients remain undertreated [15]–[19]. Therefore, the integrated STEMI care program we developed and implemented will include pre-hospital and in-hospital care. As preliminary data looks promising, we must keep improving all points of the health care system.

### Study limitations

This single center registry should be combined with the registries of other receiving centers in the city to know the real STEMI profile in Jakarta. However, our center is the cardiac referral hospital in Jakarta with the highest case load, thus characteristics of the patients in our National Cardiovascular Center Harapan Kita registry will reflect the STEMI profile in Jakarta very well. Furthermore, this study provides preliminary data with comparatively low power.

### Conclusion

For STEMI patients, the introduction of a regional AMI network has significantly increased the number of inter-hospital referral cases and the number of patients who underwent acute reperfusion procedures in the receiving center, with more patients who reached door-to-needle time <30 minutes. However, the proportion of patients who presented very late, the door-to-balloon time, and the in-hospital mortality have not improved. The receiving and referral center protocols have to be adapted to increase the quality of care of AMI patients in Jakarta.

## Supporting Information

Figure S1 **The pre-hospital triage of AMI patients in Jakarta Cardiovascular Care Unit Network System.** An internet-based ECG transmission system (Heart line) is located in the Emergency Department of the National Cardiovascular Center Harapan Kita Hospital with 24 hours service. Diagnosis and choice of reperfusion therapy will be decided through the Heart line. The choice of fibrinolytic agent is either Streptokinase or Alteplase. In post-fibrinolytic patients, rescue PCI will be performed if fibrinolysis has failed. After a successful fibrinolytic therapy, coronary angiography will be performed within 3–24 hours. EMS = emergency medical service, BP = blood pressure, HR = heart rate, RR = respiratory rate, SR = sinus rhythm, SB = sinus bradycardia, ST = sinus tachycardia, AF = atrial fibrillation, SVT = supra-ventricular tachycardia, VT = ventricular tachycardia, VF = ventricular fibrillation, AV = atrioventricular, NCCHK = national cardiovascular center Harapan Kita, RBBB = right bundle branch block, LBBB = left bundle branch block, PPCI = primary percutaneous coronary intervention, FMC = first medical contact, p.o = per os (oral).
(DOCX)

Figure S2 **The communication form and fibrinolytic check list for the emergency medical service/ambulance staff.** STEMI = ST-segment elevation myocardial infarction, non STE ACS = non-ST elevation acute coronary syndrome, CNS = central nervous system, AV = arteriovenous, BP = blood pressure, NCCHK = National Cardiovascular Center Harapan Kita.
(DOCX)
