# Supplementary material for: Temporal Trends of System of Care for STEMI: Insights from the Jakarta Cardiovascular Care Unit Network System
Source: PLoS One. 2014 Feb 10;9(2):e86665. doi: 10.1371/journal.pone.0086665 (PMC3919720; doi:10.1371/journal.pone.0086665)
Supplement: Figure S1 — The pre-hospital triage of AMI patients in Jakarta Cardiovascular Care Unit Network System. An internet-based ECG transmission system (Heart line) is located in the Emergency Department of the National Cardiovascular Center Harapan Kita Hospital with 24 hours service. Diagnosis and choice of reperfusion therapy will be decided through the Heart line. The choice of fibrinolytic agent is either Streptokinase or Alteplase. In post-fibrinolytic patients, rescue PCI will be performed if fibrinolysis has failed. After a successful fibrinolytic therapy, coronary angiography will be performed within 3–24 hours. EMS = emergency medical service, BP = blood pressure, HR = heart rate, RR = respiratory rate, SR = sinus rhythm, SB = sinus bradycardia, ST = sinus tachycardia, AF = atrial fibrillation, SVT = supra-ventricular tachycardia, VT = ventricular tachycardia, VF = ventricular fibrillation, AV = atrioventricular, NCCHK = national cardiovascular center Harapan Kita, RBBB = right bundle branch block, LBBB = left bundle branch block, PPCI = primary percutaneous coronary intervention, FMC = first medical contact, p.o = per os (oral). (DOCX) [file pone.0086665.s001.docx]

Name: Mr/Mrs/Ms.

Date of Birth:

Referral center name and location:

Medical history:

Medication history:

Times hr : min

Symptom start :

Call for medical help :

Ambulance/EMS arrival :

Ambulance departure :

Self transportation

departure :

Arrival at hospital :

ECG transmission :

Physical examination: Sens: BP /

HR x/min RR x/min

Rhythm: SR/ST/SB/AF/SVT/VT/VF/AV Block

A patient with chest discomfort

12 lead ECG

ST segment elevation:

inferior: II, III, aVF

lateral: I, aVL, V5-V6

anterior: V2-V4

extensive anterior: I,aVL-V1-V6

posterior: V7-V9 or ST in V1-V2

RBBB, LBBB,

pacemaker rhythm

Normal ECG

Symptom ≤ 12 hours

Refer to community hospital

Symptom > 12 hours

Refer to receiving centers (PCI center) for evaluation

Transmit ECG through Heart line at NCCHK

Aspirin 160-320 mg p.o

Clopidogrel 300-600 mg

Aspirin 160-320 mg p.o

Clopidogrel 300-600 mg p.o

Refer to receiving centers (PCI center) for PPCI, if FMC to balloon < 120 min, otherwise fibrinolytic during transportation

**Figure S1.**
