# Supplementary material for: Temporal Trends of System of Care for STEMI: Insights from the Jakarta Cardiovascular Care Unit Network System
Source: PLoS One. 2014 Feb 10;9(2):e86665. doi: 10.1371/journal.pone.0086665 (PMC3919720; doi:10.1371/journal.pone.0086665)
Supplement: Figure S2 — The communication form and fibrinolytic check list for the emergency medical service/ambulance staff. STEMI = ST-segment elevation myocardial infarction, non STE ACS = non-ST elevation acute coronary syndrome, CNS = central nervous system, AV = arteriovenous, BP = blood pressure, NCCHK = National Cardiovascular Center Harapan Kita. (DOCX) [file pone.0086665.s002.docx]

Date: __ /__ /____ First ambulance call: ____:____ Ambulance ID:

**Patient’s information:**

Name:

Date of birth/age:__ __ ____/ _____ years

Body weight:______ kg

Start of symptom: ___ : ___

Diagnosis: STEMI Non-STE ACS Non cardiac

First ECG transmitted:____:____

Call Heart line: ___ : ___

Estimated duration of arrival at receiving center (PCI center): ________ minutes

Receiving center destination:

**Fibrinolytic check list:**

Did the patient have:

- previous intracranial hemorrhage or stroke ? Yes/No

- ischaemic stroke in the preceding 6 months? Yes/No

- CNS damage or neoplasms or AV malformation? Yes/No

- recent major trauma/surgery/head injury? Yes/No

- gastrointestinal bleeding within the past month? Yes/No

- known bleeding disorder? Yes/No

- aortic dissection? Yes/No

- non compressible punctures in the past 24 hours? Yes/No

- transient ischemic attack in the last 6 months? Yes/No

- received oral anticoagulant therapy? Yes/No

- systolic BP > 180 mmHg or diastolic BP > 110 mmHg? Yes/No

- advanced liver disease? Yes/No

- infective endocarditis? Yes/No

- active peptic ulcer? Yes/No

- prolonged or traumatic resuscitation Yes/No

If all questions are answered with NO, fibrinolytic therapy could be given. Consult to Heart line team at NCCHK before starting fibrinolytic therapy.

Time of fibrinolytic started: ____ : ____

Fibrinolytic agent: Streptokinase/Alteplase

**Figure S2.**
